# Barriers to timely nutritional intervention in ICU patients: exploring predictive factors and neuroendocrine regulatory pathways

**DOI:** 10.3389/fnut.2025.1653688

**Published:** 2025-09-04

**Authors:** Jiali Liu, Yali Xu, Yun Xu, Jiawen Chen, Jianfeng Duan, Minhua Cheng, Wenkui Yu

**Affiliations:** ^1^Department of Critical Care Medicine, Nanjing Drum Tower Hospital, Affiliated Hospital of Medical School, Nanjing University, Nanjing, Jiangsu, China; ^2^State Key Laboratory of Pharmaceutical Biotechnology, Department of Critical Care Medicine, Nanjing Drum Tower Hospital, Nanjing University, Nanjing, China; ^3^Department of Critical Care Medicine, Nanjing Drum Tower Hospital, Drum Tower Clinical College, Nanjing University of Chinese Medicine, Nanjing, China

**Keywords:** critically ill patients, nutritional support, inflammatory response, hypercatabolism, epigenetic modification

## Abstract

To elucidate determinants of suboptimal early nutritional support achievement in critical illness. 403 adult ICU patients receiving nutritional support during days 1–7 of admission were enrolled in this retrospective study. For patients, basic patient information, disease severity, inflammatory indicators, and prognostic indicators were collected to explore the reasons for the poor rate of early nutritional support. In addition to observing ICU patients, adult male SD rats were injected with LPS dissolved in saline (10 mg/kg) via a single intraperitoneal injection to simulate the inflammatory state caused by human infection. Skeletal muscle tissues and hypothalamic tissues of rats at different time point (6, 12, and 24 h) s were taken for methylation assay, respectively. The baseline APACHE II (24.45 vs. 21.17, *p* < 0.001) and CRP (126.44 vs. 88.00 mg/L, *p* < 0.001) were significantly higher in the non-achieving group (failed to meet 80% target calories by day 7) than in the target-achieving group. Inverse correlations existed between caloric delivery and inflammatory markers (WBC: *r* = −0.313; CRP: *r* = −0.311). Septic rats exhibited time-dependent hypothalamic promoter methylation changes absent in skeletal muscle. Systemic inflammation and disease severity constitute primary barriers to nutritional target achievement, potentially mediated through central epigenetic regulation.

## 1 Introduction

The persistent challenge of optimizing nutritional support in critical illness reflects the tension between physiological needs and metabolic constraints. Currently, there are still controversies about the timing and dosage of nutritional support for critically ill patients. Current guidelines recommend initiating enteral nutrition (EN) within 24–48 h of intensive care unit (ICU) admission, with 80–100% of target calories achieved by days 3–7 (1, 2). However, the current target attainment rate is still far from satisfactory. It has been reported that the rate of critically ill patients reaching the target calories within a week range from 20 to 70% (3–8). A recent cross-sectional study by Weiqin Li et al. showed that in 2017, only 17.8% of Chinese adult ICU patients met target calories within 1 week (9). The persistent gap between guidelines and practice raises two questions: (1) Are implementation barriers (e.g., timing, dosing) responsible? (2) Do patient-specific factors, such as disease severity or metabolic states, limit nutritional target achievement?

Hypercatabolism, driven by systemic inflammation, is a proposed mechanism for impaired nutrient utilization in critical illness (10, 11). Our previous work implicates hypothalamic nuclei (particularly the arcuate nucleus) as potential orchestrators of this metabolic disruption (12–16), suggesting a paradigm where central epigenetic modifications may coordinate peripheral metabolic responses to systemic inflammation.

Based on the above problems, we intend to review the target calories attainment rate of nutritional support for critically ill patients in the early stage (within 7 days) under the standardized nutritional support process (17), as described in the Supplementary materials. Next, we analyze the influencing factors affecting the nutritional support attainment rate, in order to clarify whether the characteristics of the critically ill patients’ diseases per se that led to failure of nutritional support to attain the target calories attainment rate.

Furthermore, through animal experiments, we will observe the changes of DNA methylation and transcriptome in hypothalamus (representative of central regulation) and skeletal muscle tissue (representative of peripheral tissues and organs) under acute stress, so as to explore whether the key regulatory points of acute metabolic changes in critically ill patients are located in the central or peripheral tissues and organs. It will provide a new solution and direction to improve the target calorie-reaching rate of early nutritional support for critically ill patients.

## 2 Materials and methods

### 2.1 Clinical trial

#### 2.1.1 Study design

Study design: a retrospective, single-arm, open-label.

488 critically ill patients admitted to the ICU ward of Nanjing Drum Tower Hospital from January 2019 to December 2019 were intended to be included. Inclusion criteria: (1) enteral or parenteral nutrition support was used within 7 days after admission; (2) ≥18 years old; (3) hospital stay ≥7 days. Exclusion criteria: (1) pregnancy and lactation; (2) patients with chronic diseases such as diabetes; (3) patients with severe organic disease in the past; (4) patients with hospitalization duration over 3 months; (5) for patients admitted to the ICU multiple times, only data of the first admission was recorded.

Primary outcome: nutritional support attainment on the 7th day.

#### 2.1.2 Data collection

Collection contents include: basic information of patients, including gender, age, height, weight, length of stay, main disease diagnosis, disease severity indicators, including the day of check-in sequential organ failure (SOFA) score, Acute Physiology and Chronic Health status score System II (APACHE II) score, Acute Gastrointestinal Injury (AGI) rating. Nutritional support indexes included nutritional support and caloric intake at 1–7 days after ICU admission. Inflammatory response indexes included white blood cell count (WBC), neutrophil ratio, C-reactive protein (CRP), and Procalcitonin (PCT) at ICU 1–7 days after ICU admission. The prognostic measures included duration of mechanical ventilation, length of stay (LOS) in ICU, and outcome (28-day mortality rate).

#### 2.1.3 Definition

In this study, we referred to the ICU nutritional support protocol of our institution, i.e., the standardized nutritional support process, and international authoritative clinical guidelines, setting the target for caloric intake at 80% of 25 kcal/kg/day by day 7. According to whether the patients reached the target on the 7th day, the enrolled patients were divided into the target-achieving group and the non-achieving group.

#### 2.1.4 Relevant indicators and calculations

The target calorie is calculated by a simplified prediction formula of 25 kcal/kg/d, where the weight is the ideal weight, which refers to the weight calculated according to the height of the patient.

Infusion rate = actual daily nutritional infusion calories/target calories ×100%. When calculating the actual infusion volume, we refer to both nursing records and medical records. Nursing records regularly record the patient’s fluid intake and output. Due to the implementation of a standardized feeding process, enteral/parenteral nutrition intake is recorded every 4 h to record the actual nutritional infusion volume and any interruptions.

Target-achieving rate = number of achievers/total number of enrolled patients ×100%.

#### 2.1.5 Statistical analysis

SPSS 26.0 statistical software was used for statistical analysis. The measurement data were described by mean ± standard deviation for normal distribution, and by median and interquartile range (IQR) for non-normal distribution. The counting data are described in frequency and percentage terms. T-test or variance analysis were used for inter-group comparison; the categorical variables were the Chi-square test or rank-sum test. The Kruskal-Wallis test was used for variables with non-normal distribution. The Wilcoxon signed-rank test was used for paired samples. Logistic regression was used to analyze the factors affecting the patients’ nutritional treatment attainment.

### 2.2 Animal experiments

#### 2.2.1 Study animals

Adult male Sprague–Dawley (SD) rats (250 ± 20 g) were purchased from the Animal Research Center of Nanjing Jinling Hospital, and the experimental rats were raised in a standard environment with regular lighting (12 h: 12 h day and night), constant temperature, adequate water, and physical objects. The experimental scheme was approved by Nanjing University Medical School Affiliated Nanjing Drum Tower Hospital Medical Ethics Committee.

#### 2.2.2 Study design

Before conducting any experiments, all rats were fed for more than 7 days to acclimate, and then 10 SD rats were randomly divided into two groups: Sepsis group (n = 9) and control group (n = 1), the rats in sepsis group were injected intraperitoneally with Lipopolysaccharide (LPS) (10 mg/kg), and the rats in control group were injected intraperitoneally with the same volume of normal saline. The rats in the sepsis group were then randomly divided into 3 groups. The rats in the 6 h, 12 h, and 24 h groups were euthanized at the corresponding time points, and the skeletal muscle tissues (extensor digitorum longus and gastrocnemius muscle) and hypothalamus tissues were taken and frozen at −80 °C.

#### 2.2.3 Methylation

A high-purity PCR preparation kit was used to obtain DNA samples, and all operations were carried out according to the instructions. The concentration and purity of the obtained DNA samples were determined using the Nanodrop 2000c spectrophotometer. Each DNA sample was sulfited using the EZ DNA methylation kit. To determine the methylation profile of the sample, methylation-specific Real-time PCR was performed using Roche LightCycler 480 II and its preset primers. CpGenome™ Rat methylated Genomic DNA Standard and CpGenome™ Rat Unmethylated Genomic DNA Standard (Merck Millipore; Burlington, MA, USA) as a positive control. The methylation results were analyzed using Light-Cycler 480 software. The methylated or unmethylated profile was identified by the TM difference in the methylated and unmethylated primers in each sample.

#### 2.2.4 RNA extraction, cDNA synthesis and RT-PCR

RNA was isolated from skeletal muscle samples of the hypothalamus using the TriPure kit. All operations were performed according to the instructions. The TriPure solution was added to each sample and homogenized with a homogenizer. The homogenized sample was added with 200 μL chloroform and incubated at room temperature for 5 min. After centrifugation (12,000 rpm, 20 min, 4 °C), the upper transparent mixture was transferred to the new tube, and isopropyl alcohol was added. The samples were incubated at room temperature for 10 min and centrifuged at 4 °C, 12,000 rpm for 10 min. Discard the supernatant and add 75% ethanol to each particle. After centrifugation, the excess ethanol in the sample was evaporated at 4 °C for 5 min at 7500 g, and the sample was re-suspended in water without RNA nuclease. The Nanodrop 2000c spectrophotometer is used to determine the concentration and purity of extracted RNA samples. For all RNA samples, the A260/A280 absorbance ratio is set between 1.8 and 2.0. cDNA was synthesized from RNA samples using the RevertAid RT reverse transcription kit (Thermo Scientific, USA). Real-Time PCR analysis was performed using the LightCycler®480 SYBR Green I Master on the Roche LightCycler 480 II device. The data after Real-Time PCR analysis were analyzed by the absolute quantitative method and the advanced relative quantitative method. For the relative quantification between results, the Ct values of target genes were normalized by ACTB. The fold change (< 0.5, significantly down-regulated and > 2, significantly up-regulated) values were calculated proportionally with the normalized values of the control group.

## 3 Results

### 3.1 Basic characteristics of clinical observations

**Figure 1:**
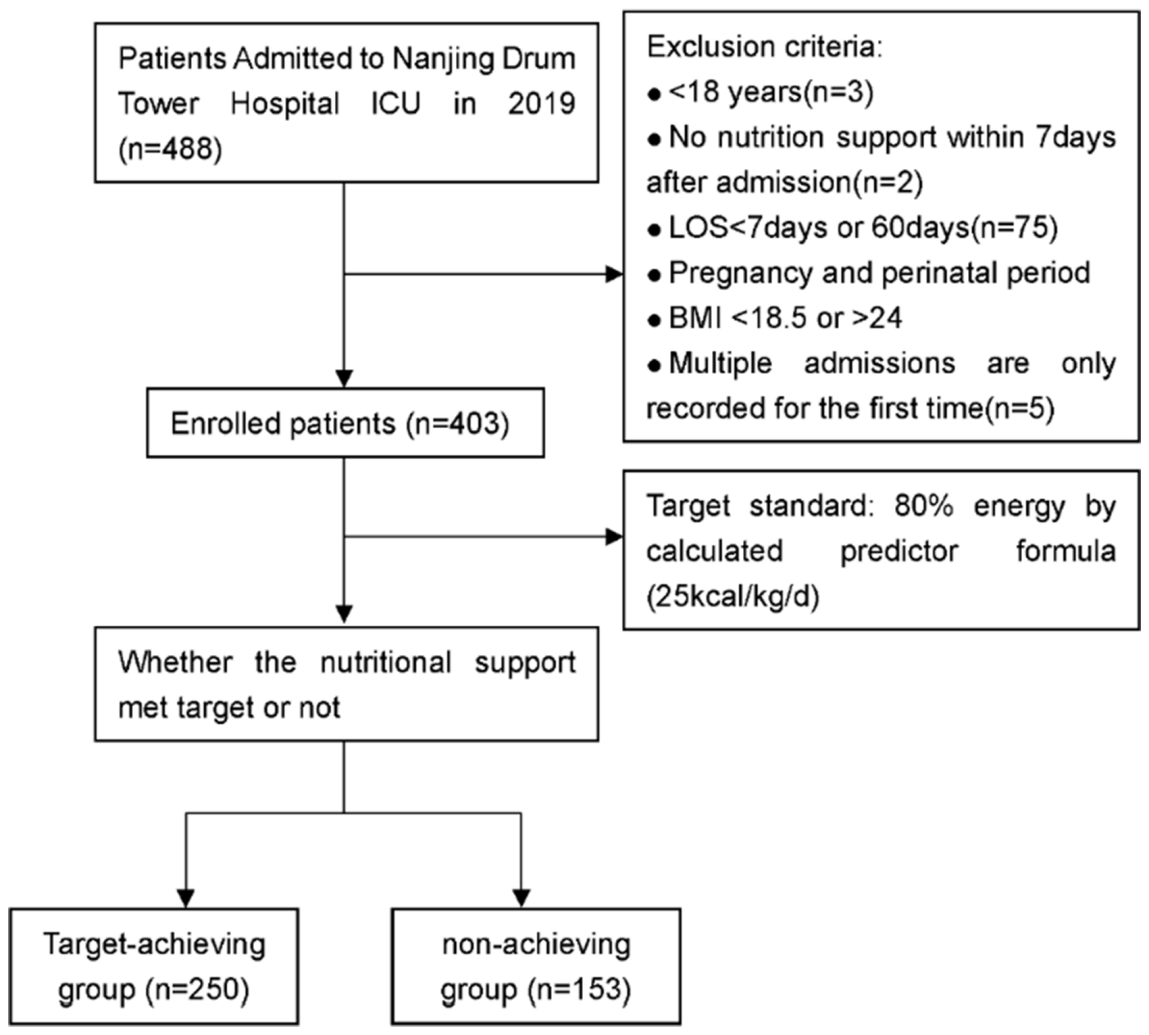
Clinical trial flow.

A total of 403 critically ill patients was included in the study, of which 249 were male (61.80%) and 154 were female (38.20%). The minimum age was 18 years, the maximum 95 years, and the median 64 years. In terms of admission types, 30 patients (7.40%) were postoperative, 69 (17.10%) trauma, 306 (75.90%) internal medicine, 221 (54.80%) respiratory diseases, 94 (23.30%) circulatory diseases, and 145 (36.00%) sepsis. The Clinical Trial Flow is shown in Figure 1.

### 3.2 Comparison of the basic situation of patients admitted to ICU between the target-achieving group and the non-achieving group

All of the 403 enrolled patients, 250 (62.00%) achieved the nutritional target by day 7, while 153 (38.00%) did not meet the standard. The demographic characteristics of the target-achieving group and the non-achieving group are shown in the Table 1. No statistically significant differences were observed in age, sex distribution, or height between groups (all *p* > 0.05). However, Patients who achieved nutritional targets had significantly higher actual body weight and BMI (*p* < 0.001). In terms of admission categories, the proportion of trauma patients in the target-achieving group (20.00%) was higher than that in the non-achieving group (12.40%), *p* = 0.05, and the rest had no significant difference. The median and interquartile distance of basic APACHE II scores in the target-achieving group were 21.17 (10) vs. 24.45 (11), respectively, *p* < 0.001. In terms of inflammation indicators, the basal CRP in the target-achieving group was significantly lower than that in the non-achieving group [88.00 (71.60) vs. 126.44 (85.60), *p* < 0.001]. Although there was no significant difference in the basic WBC count of patients admitted to the ICU between the two groups, the proportion of abnormal WBC in the target-achieving group (59.60%) was significantly lower than that in the non-achieving group (77.10%), *p* < 0.001 (Table 1).

**Table 1:** Comparison of the basic situation of patients admitted to ICU between the target-achieving group and the non-achieving group.

Variables	Total	Target-achieving group	Non-target-achieving group	*p*
*N* (%)	403	250 (62.00)	153 (38.00)	
Age median (IQR)	64.04 (23)	64.34 (23)	63.63 (20)	0.583
Sex				
Male *N* (%)	249 (61.80)	154 (61.60)	95 (62.10)	
Female *N* (%)	154 (38.20)	96 (38.40)	58 (37.90)	
Height (cm) median (IQR)	172.71 (12)	173.08 (11)	172.41 (13)	0.483
Weight (kg) median (IQR)	65.61 (15)	67.26 (12)	63.37 (16)	<0.001
BMI (kg/㎡) median (IQR)	21.86 (2.52)	22.36 (1.81)	21.13 (2.49)	<0.001
Ideal Weight (kg) median (IQR)	67.71 (12)	68.08 (11)	67.41 (13)	0.483
Target energy(kcal/d) median (IQR)	1692.82 (300)	1701.88 (281)	1685.31 (325)	0.483
Admission type				
Surgery *n* (%)	30 (7.40)	19 (7.60)	11 (7.20)	0.879
Trauma *n* (%)	69 (17.10)	50 (20.00)	19 (12.40)	0.050
Internal medicine^1^ *n* (%)	306 (75.90)	183 (73.20)	123 (80.40)	0.101
Respiratory *n* (%)	221 (54.80)	132 (52.80)	89 (58.20)	0.293
Circulation *n* (%)	94 (23.30)	57 (22.80)	37 (24.20)	0.750
Sepsis *n* (%)	145 (36.00)	63 (25.20)	82 (53.60)	<0.001
Severity of illness				
APACHE II median (IQR)	22.45 (11)	21.17 (10)	24.45 (11)	<0.001
SOFA median (IQR)	7.68 (6)	7.60 (6)	7.75 (6)	0.670
Laboratory tests at admission				
WBC/10^9^, median(IQR)	12.93 (7.50)	12.47 (7.30)	13.12 (7.70)	0.153
Neutrophil, n% median (IQR)	0.87 (0.08)	0.87 (0.09)	0.86 (0.08)	0.416
CRP, median(IQR)	103.14 (81)	88.00 (71.6)	126.44 (85.6)	<0.001
WBC abnormity n (%)	267 (66.30)	149 (59.60)	118 (77.10)	<0.001

^1^Internal medicine, covers all kinds of diseases that are mainly treated with internal medicine, except for those related to surgery; Respiratory, respiratory system diseases; Circulatory, circulatory system diseases.

### 3.3 Comparison of the level of inflammatory response between the target-achieving group and the non-achieving group

**Figure 2:**
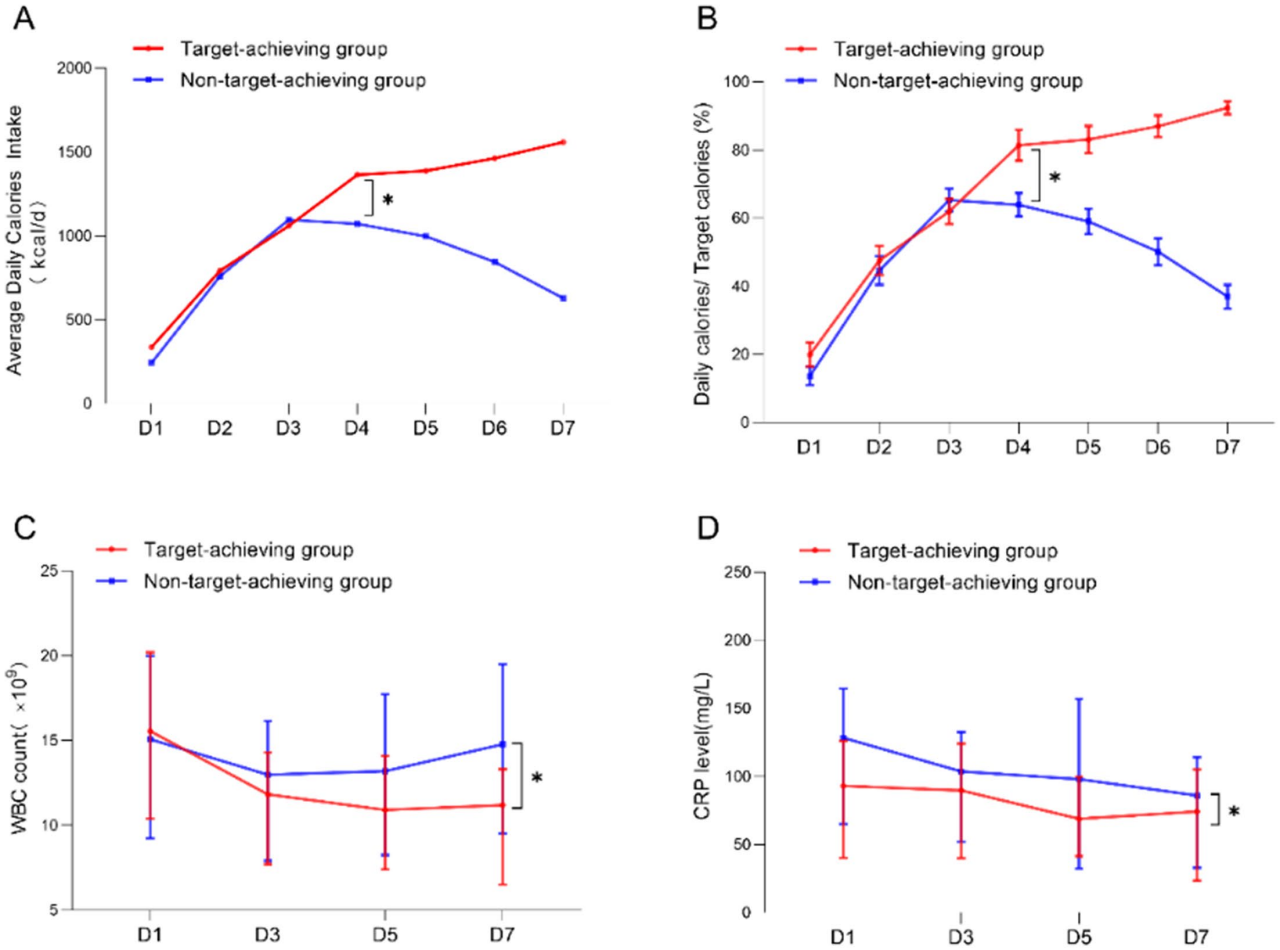
Comparison of the level of inflammatory response between the target-achieving group and the non-achieving group. **(A,B)** Average daily calorie intake and daily infusion in the target-achieving and the non-achieving groups; **(C,D)** dynamics of WBC and CRP on days 1 and 7 in the target-achieving and the non-achieving groups.

We conducted a comparative analysis of 7-day nutritional infusion parameters and inflammatory markers between the two groups. During the first 3 days, both the target-achieving and non-achieving groups showed comparable caloric intake and infusion rates (all *p* > 0.05), confirming equivalent initial nutritional support in both groups. However, beginning on day 4, the non-achieving group demonstrated a progressive decline in both caloric delivery and infusion rate, showing statistically significant differences from the target-achieving group (Figures 2A,B). Serial measurements of WBC and CRP levels revealed distinct temporal patterns: while inflammatory markers progressively decreased in the target-achieving group, they remained persistently elevated in the non-achieving group throughout the observation period (Figures 2C,D), This differential inflammatory response likely contributed to the failure to achieve nutritional targets in the non-achieving group by day 7. These findings collectively suggest that the initial systemic inflammatory response and hypercatabolic state in critically ill patients may limit early nutritional tolerance. Our data support prioritizing inflammatory control before attempting aggressive nutritional support, as the metabolic milieu during acute illness appears to determine nutritional feasibility. The observed divergence in outcomes after day 3 may represent a critical transition point in the disease course where inflammatory status dictates nutritional tolerance.

### 3.4 Association between inflammatory status and nutritional delivery efficiency

**Figure 3:**
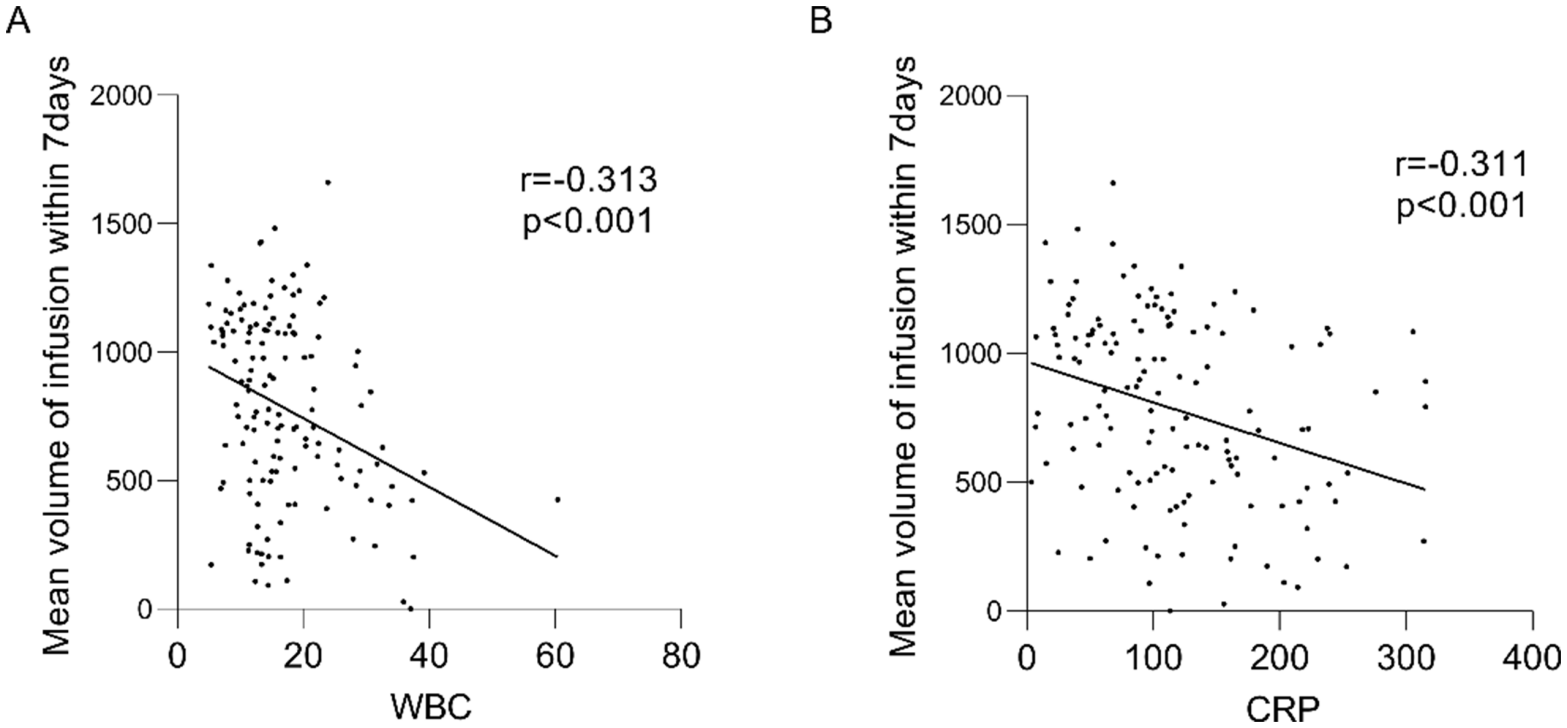
Mean volume of infusion within 7 days correlates with WBC, CRP. **(A)** Mean infusion volume within 7 days correlated with WBC; **(B)** Mean infusion volume within 7 days correlated with CRP. r: correlation coefficient.

Our correlation analyses revealed significant inverse relationships between inflammatory markers and nutritional delivery: the 7-day average infusion volume showed negative correlations with both WBC and CRP levels (Figure 3). Additionally, we observed that daily nutritional intake was associated with the preceding day’s SOFA score (Table 2). Based on the above results, we speculate that the severity of systemic inflammation and the overall severity of the disease both have a significant impact on the nutritional support capacity of critically ill patients. Notably, while all patients received comparable nutritional support during the initial phase, those with more severe inflammation and higher illness severity showed progressively impaired nutrient utilization. This suggests the existence of a physiological threshold for nutritional tolerance, likely mediated through central regulatory mechanisms that prioritize endogenous energy mobilization (e.g., muscle catabolism) during severe systemic inflammation. In the context of severe inflammation, the correlation between nutritional intake and adverse outcomes warrants caution. The mechanism may be limited metabolic capacity, but this hypothesis needs to be verified by future research.

**Table 2:** Correlation of infusion rates with disease severity scores, inflammation indicators.

	Daily infusion rate	Daily infusion volume	Infusion rate next day	Infusion volume next day
APACHEII score				
D1	0.010 (0.849)	0.009 (0.866)	−0.076 (0.138)	−0.065 (0.204)
D4	−0.011 (0.829)	0.006 (0.898)	−0.024 (0.635)	−0.005 (0.921)
D7	−0.060 (0.235)	−0.035 (0.488)		
SOFA score				
D1	−0.056 (0.274)	−0.056 (0.276)	−0.212** (0.000)	−0.210** (0.000)
D4	−0.195** (0.000)	−0.208** (0.000)	−0.153** (0.002)	−0.171** (0.001)
D7	−0.026 (0.602)	−0.018 (0.726)		
WBC				
D1	−0.099* (0.047)	−0.102* (0.041)	−0.183** (0.000)	−0.189** (0.000)
D3	−0.119* (0.020)	−0.122* (0.016)	−0.066 (0.195)	−0.071 (0.165)
D5	−0.163** (0.001)	−0.148** (0.004)	−0.118* (0.021)	−0.100 (0.053)
D7	−0.174** (0.000)	−0.143** (0.004)		
Neutrophil				
D1	−0.101* (0.042)	−0.100* (0.044)	−0.105** (0.035)	−0.102* (0.042)
D3	−0.181** (0.000)	−0.172** (0.001)	−0.063 (0.214)	−0.067 (0.190)
D5	−0.033 (0.516)	−0.040 (0.442)	−0.027 (0.605)	−0.031 (0.545)
D7	0.048 (0.364)	0.052 (0.324)		
CRP				
D1	−0.173** (0.000)	−0.184** (0.000)	−0.186** (0.000)	−0.201** (0.000)
D3	−0.291** (0.000)	−0.293** (0.000)	−0.187** (0.001)	−0.203** (0.000)
D5	−0.176** (0.001)	−0.166** (0.002)	−0.081 (0.138)	−0.069 (0.210)
D7	−0.148** (0.0030)	−0.143** (0.004)		
CRP grade(D7)	−0.155** (0.002)	−0.151** (0.002)		
WBC grade(D7)	−0.215** (0.000)	−0.199** (0.000)		

* *p* < 0.05; ** *p* < 0.001.

### 3.5 Stratified analysis of nutritional delivery in non-achieving patients

To better understand the determinants of nutritional support success, we performed a stratified analysis of the non-achieving group (*n* = 153), categorizing patients into four subgroups based on their actual nutrition delivery proportion relative to targets: 0–20, 20–40%, 40–60% and 60–80% achievement. The 28-day mortality rate, WBC, CRP, APACHE II score, SOFA score, and length of ICU stay among the four groups were compared. No statistically significant differences in inflammatory markers, or other measured parameters across the four subgroups (Table 3). We also noted a paradoxical trend toward higher mortality with increasing nutrition delivery rates (Table 4). The observed inverse mortality trend warrants further investigation into potential risks of aggressive nutrition in certain critically ill populations.

**Table 3:** The WBC, CRP, APACHE II score, SOFA score and length of hospital stay were compared among the four groups.

	WBC (D7)	WBC variation	CRP (D7)	CRP variation	SOFA score(D7)	SOFA Score variation	APACHEII score (D7)	APACHEI score variation	LOS
Mann-Whitney U	2612.00	2391.50	2697.00	2553.00	2424.50	2772.50	2518.50	2494.50	2854.50
Wikerson W	6449.00	4602.50	4908.00	4764.00	4504.50	4983.50	4598.50	6322.50	6682.50
Z	−0.921	−1.766	−0.641	−1.171	−1.024	−0.364	−0.657	−1.388	−0.061
Significance (two-tailed)	0.357	0.077	0.522	0.241	0.306	0.716	0.511	0.165	0.951

**Table 4:** Comparison of mortality between four groups.

		0–20%	20–40%	40–60%	60–80%
Mortality	count	12 (43)	5 (23)	11 (30)	22 (57)
	percentage	27.9%	21.7%	36.7%	38.6%

### 3.6 Central regulatory mechanism of hypercatabolism in sepsis

**Figure 4:**
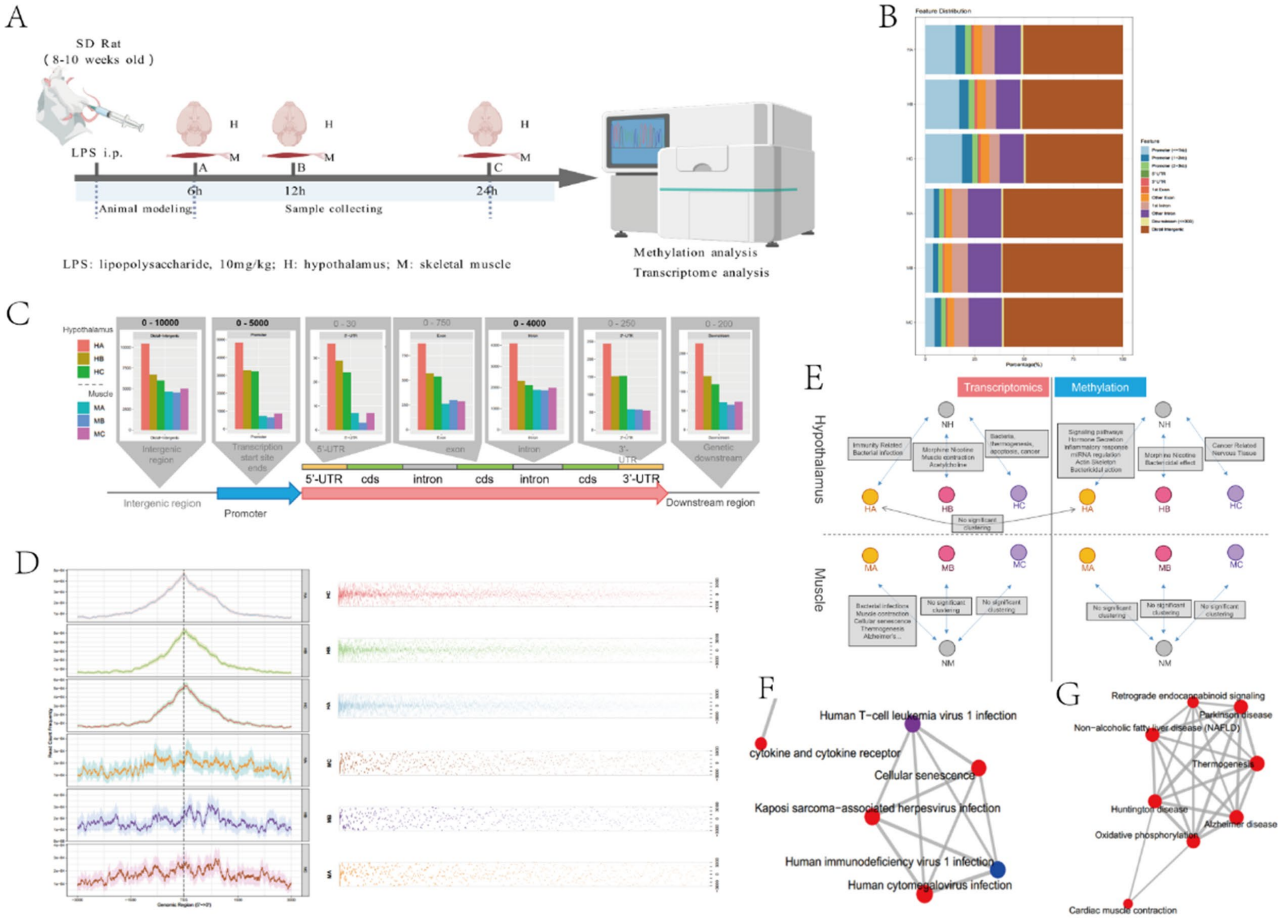
Hypothalamus and skeletal muscle methylation results. **(A)** The experimental design of rat models with different infection times. This model was created by BioGDP.com (34). **(B)** Analysis of differentially methylated expression regions in hypothalamus and skeletal muscle tissues; **(C)** Relationship between different time and differentially methylated regions in hypothalamus and skeletal muscle tissues; **(D)** Positional analysis of differentially methylated regions in hypothalamus and skeletal muscle; **(E)** Analysis of transcriptome and methylation enrichment in hypothalamus and skeletal muscle tissues; **(F)** Clustering of bacterial infection pathways in muscle MA; **(G)** Muscle contraction pathway clustering in MA.

These findings collectively indicate that the severity of illness and intrinsic metabolic disorders in critically ill patients are closely related to whether nutritional goals are achieved. Substantial evidence confirms that critical illness induces a profound hypercatabolic state, characterized by two hallmark features: (1) suppressed nutrient intake and (2) accelerated skeletal muscle proteolysis, as consistently observed in our prior experimental sepsis models. The hypothalamic regulation of this metabolic derangement has been mechanistically established through several lines of evidence (12–16, 18, 19). To further elucidate the central regulatory mechanisms underlying sepsis-induced hypercatabolism, we conducted comprehensive molecular profiling comparing septic and control rats (Figure 4A). Methylation and transcriptome analysis were performed on hypothalamus and skeletal muscle tissue of sepsis and control rats at different time points, aiming to verify the central mechanism of sepsis hypercatabolism at the gene expression level.

Our genome-wide methylation analysis revealed distinct epigenetic patterning between hypothalamic and skeletal muscle tissues in septic rats (Figure 4B). Quantitative comparisons demonstrated three key tissue-specific methylation patterns: (1) Hypothalamic tissue exhibited a greater number of differentially methylated regions (DMRs) in promoter regions compared to skeletal muscle (*p* < 0.01); (2) Conversely, skeletal muscle demonstrated predominant methylation alterations in distal intergenic regions, showing more DMRs than hypothalamic tissue (*p* < 0.05); (3) No statistically significant differences were observed in other genomic regions (5’UTR, 3’UTR, exons, or introns) between tissue types (all *p* > 0.05) (Figure 4B). Temporal analysis (Figure 4C) demonstrated that hypothalamic methylation levels exhibited progressive changes across three experimental timepoints (6 h, 12 h, 24 h post-LPS), showing strong correlation with duration of sepsis. Notably, while promoter-associated DMRs were abundant in hypothalamic tissue, skeletal muscle displayed no significant time-dependent methylation changes. Spatial distribution patterns (Figure 4D) revealed two distinct organizational principles: (1) Hypothalamic DMRs showed significant clustering within specific genomic loci, particularly enriched near transcription start sites (TSS); (2) Skeletal muscle DMRs maintained uniform genomic distribution. This differential spatial organization suggests that hypothalamic methylation changes likely represent targeted regulatory modifications, whereas skeletal muscle alterations may reflect generalized epigenetic disturbances.

In addition, we conducted enrichment analysis of methylation and transcriptome in the hypothalamus and skeletal muscle tissue, respectively. The results were shown in Figures 4E–G. In muscle tissue, the transcriptome mainly clustered the expression of genes such as bacterial infection, muscle contraction, cell aging, and thermogenesis, while no significant methylation clustering was found. In the hypothalamus, methylation was mainly related to the expression of genes such as neuroendocrine regulation, hormone secretion, inflammatory response, miRNA regulation, and actin skeleton, while transcriptome was mainly related to the expression of genes such as bacterial infection, immune response, muscle contraction, thermogenesis, and apoptosis (Figure 4E).

Integrating these findings, we propose a mechanistic model wherein the hypothalamus orchestrates sepsis-induced metabolic dysregulation through epigenetic reprogramming. Specifically, our data demonstrate that: (1) sepsis triggers selective methylation alterations in hypothalamic gene promoters (particularly near transcription start sites); (2) these epigenetic modifications directly influence the expression of neuroendocrine regulators (e.g., POMC, AgRP); (3) the consequent hypothalamic signaling drives peripheral metabolic adaptations, including the observed skeletal muscle catabolism. This central-peripheral regulatory axis represents a fundamental mechanism underlying the hypercatabolic state in sepsis. While our current findings identify key genomic regions and functional pathways involved (neuroendocrine regulation, inflammatory response, etc.), several critical questions remain unresolved: (1) the precise identity of methylation-sensitive hypothalamic effector genes; (2) the complete signaling cascades connecting central epigenetic changes to peripheral tissue metabolism; and (3) potential therapeutic targets within this regulatory network. Future studies will be essential to fully elucidate these mechanisms.

## 4 Discussion

EN remains a cornerstone of metabolic support in critical care. Since the implementation of standardized EN protocols in 2019, we have observed significant improvements in nutritional support outcomes for critically ill patients. However, persistent challenges remain in achieving recommended caloric targets, with only 59.2% of patients meeting 80% of energy requirements within 7 days - a rate comparable to international reports yet substantially below ESPEN guidelines (9, 20–22). This persistent gap underscores that protocol adherence alone cannot overcome the fundamental metabolic constraints imposed by severe inflammatory states. How to improve the tolerance upper limit of nutritional support is a problem that needs attention at present.

Current research on optimizing nutritional support in critical care predominantly emphasizes technical aspects, including delivery methods, formula composition, and feeding interruptions (23), while largely overlooking the fundamental influence of underlying disease severity. This paradigm is challenged by emerging evidence demonstrating potential benefits of restrictive nutritional strategies during acute critical illness. Reignier et al. established that moderate caloric and protein restriction correlates with improved clinical recovery (24), while Tatucu-Babet et al. identified enhanced autophagic activity with controlled underfeeding (25). These findings align with mechanistic studies attributing limited efficacy of aggressive early nutrition to three key factors: (1) acquired anabolic resistance (26) (2) impaired utilization of supplemented amino acids, and (3) suppression of endogenous repair mechanisms, including autophagy and ketogenesis (27–29). Collectively, this evidence supports a patient-tailored approach to nutritional support that dynamically adjusts to disease severity markers, particularly the magnitude of systemic inflammatory response.

This study is based on single-center retrospective data and preliminarily explores the factors affecting the achievement of nutritional targets in critically ill patients through the evaluation of clinical and molecular parameters. Our analysis revealed that protocol adherence during initial nutritional support implementation was comparable between groups, with significant divergence emerging specifically during the subacute phase (days 4–7) of critical illness. The temporal pattern of nutritional delivery (as shown in the Figures 2A,B) reveals important pathophysiological insights. Despite aggressive early EN initiation, caloric intake typically plateaus after day 4, coinciding with peak inflammatory activity. This phenomenon suggests that current nutritional strategies may need to account for the dynamic metabolic capacity during critical illness. Clinically, we also observed that 3–5 days of the disease course in critically ill patients are usually a turning point and watershed in the evolution of the disease, in alignment with the retrospective analysis results. Our data indicate that EN tolerance is intrinsically limited during periods of intense systemic inflammation, supporting a more nuanced approach than strict adherence to fixed caloric targets. This pattern also strongly suggests an association between reduced nutritional infusion and patient inflammation levels, but the influence of clinicians intentionally restricting feeding due to the severity of the patient’s condition cannot be ruled out.

The observed impaired metabolic adaptability was most strongly associated with the magnitude of systemic inflammation, as evidenced by sustained elevation of canonical biomarkers. This inflammatory milieu induces a characteristic triad of metabolic disturbances: (1) acquired insulin resistance, (2) accelerated proteolysis, and (3) appetite dysregulation - collectively constituting the hypercatabolic state (30). The pathophysiological consequences of this state create a fundamental biological constraint on nutrient utilization, explaining the inverse relationship between inflammatory activity and caloric tolerance. These findings fundamentally shift our understanding of nutritional support limitations in critical illness. The observed nutritional intolerance appears to be related to an adaptive metabolic response to severe inflammation. This paradigm suggests that therapeutic efforts should prioritize modulation of the underlying inflammatory drive rather than aggressive pursuit of caloric targets during acute physiological stress.

The results of methylation studies in rat hypothalamus suggest that epigenetic regulation of the hypothalamus may be involved in the impaired nutritional tolerance, but the causal relationship and specific mechanisms of action require further research to validate. The characteristic metabolic alterations - including accelerated muscle proteolysis and suppressed protein synthesis - reflect coordinated systemic responses more consistent with central nervous system regulation than with isolated peripheral organ dysfunction. This is substantiated by our experimental evidence demonstrating: (1) a strong correlation between skeletal muscle catabolism and hypothalamic neuropeptide expression (POMC, AgRP) in sepsis models (31); and (2) significant attenuation of muscle wasting following targeted inhibition of hypothalamic POMC signaling (14). The molecular basis of this central regulation appears to involve stress-induced epigenetic modifications. While LPS-mediated DNA methylation changes have been documented in pulmonary tissue during acute lung injury (32), our study provides novel evidence of sepsis-associated methylation alterations specifically in hypothalamic tissue. The observed tissue-specific epigenetic patterns - with predominant promoter region methylation in the hypothalamus versus distal intergenic changes in skeletal muscle - suggest a hierarchical regulatory relationship. This is further supported by transcriptomic data showing that skeletal muscle gene expression changes likely represent downstream effects of primary hypothalamic alterations. These results provide new insights into the understanding of nutritional intolerance in critically ill patients. In addition to the limitations of nutrition support techniques, the impaired nutrient utilization associated to originate from inflammation-triggered central nervous system reprogramming. This paradigm suggests that optimizing nutritional support strategies may require: (1) recognition of this central regulatory constraint during acute inflammation; and (2) development of interventions targeting hypothalamic signaling pathways. While the specific genes and mechanisms require further elucidation, our findings establish hypothalamic epigenetic regulation as a critical determinant of metabolic responses in critical illness.

Our methylation cluster analysis revealed significant impairment of muscle contractile function in septic rats, indicating that sepsis affects both muscle mass and function. We propose that sepsis-induced hypothalamic dysregulation may contribute to these cardiac metabolic alterations, similar to its effects on skeletal muscle, where emerging evidence demonstrates that septic cardiomyopathy - characterized by diastolic dysfunction — substantially worsens patient prognosis (33). The identified muscle tissue genetic modifications may suggest potential therapeutic targets for septic cardiomyopathy. However, due to species differences, these findings cannot be directly applied to ICU patients. Further investigation is needed in the future to validate these findings. Subsequent prospective experiments will collect more clinical case data to analyze the correlation between patients’ inflammatory marker levels, the epigenetic status of hypothalamus-related genes, and the effects of nutritional support. This may help explore the relationship between inflammatory markers, epigenetic marker levels, and nutritional support strategies.

This study has several methodological limitations that require attention. First, the single-center design may limit the generalizability of the study results. Future studies should expand the sample size through multicenter studies to validate the study results. Second, retrospective studies rely on past recorded data, which may be incomplete. This limitation restricts our assessment of inflammatory markers and baseline nutritional markers, potentially affecting our comprehensive evaluation of patients’ overall condition and nutritional status, leading to an incomplete understanding of certain underlying mechanisms. Additionally, as a retrospective observational study, we cannot determine the temporal relationship between active nutritional support and adverse outcomes, nor can we establish a clear causal relationship between the two. A prospective study is necessary in the future, incorporating baseline nutritional markers (albumin, prealbumin, and transferrin) and inflammatory markers into the data collection scope during the study design phase. This will enable a comprehensive assessment of patients’ nutritional status and inflammatory responses, as well as an in-depth exploration of their interrelationships and their combined impact on patient outcomes. Third, although our institution implemented standardized nutritional support process, the experimental design did not assess barriers to the implementation of nutritional strategies, which is a limitation. Future research could employ qualitative research methods, such as surveys or focus group discussions with clinicians, to further explore the impact of barriers to nutritional strategy implementation on achieving patients’ caloric requirements. Fourth, animal experiments can only simulate the inflammatory state of critically ill patients and cannot directly reflect their nutritional status. Although the experimental results indicate that sepsis is associated with a hypermetabolic state mediated by hypothalamic epigenetics, which may be related to poor nutritional tolerance, the small sample size of animal experiments limits the feasibility of translational research. Further validation is needed through larger-scale experimental groups.

This study suggests that the failure to achieve nutritional targets in critically ill patients may be primarily driven by inflammation-induced metabolic dysregulation, rather than suboptimal delivery of nutritional support. Our findings reveal a central role of hypothalamic epigenetic reprogramming in mediating the hypercatabolic state, which imposes intrinsic limitations on nutrient utilization during acute critical illness. These insights challenge the conventional emphasis on strict caloric adherence and instead advocate for an inflammation-adapted approach that prioritizes metabolic stabilization over aggressive feeding. Future research should focus on elucidating the specific hypothalamic pathways involved and developing targeted interventions to modulate central metabolic regulation, thereby improving nutritional tolerance and clinical outcomes in critically ill patients.

## Data Availability

The original contributions presented in the study are included in the article/Supplementary material, further inquiries can be directed to the corresponding authors.
