# Supplementary material for: Barriers to timely nutritional intervention in ICU patients: exploring predictive factors and neuroendocrine regulatory pathways
Source: Front Nutr. 2025 Sep 4;12:1653688. doi: 10.3389/fnut.2025.1653688 (PMC12443761; doi:10.3389/fnut.2025.1653688)
Supplement: Supplementary file 1 [file Image_1.pdf]

Supplementary Material

1 Supplementary Figures and Tables

1.1 Supplementary Figures

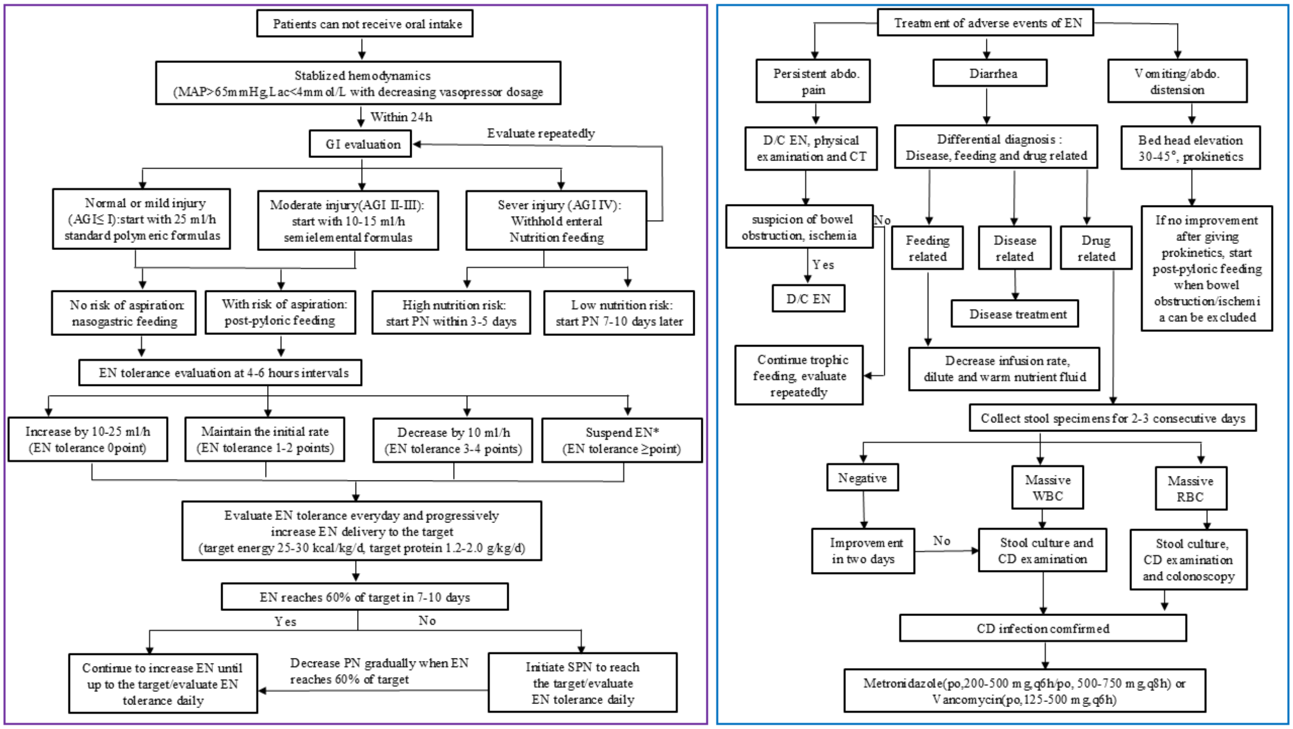

Supplementary Figure 1. Standardized feeding process flow chart. As with the previously established research protocol(1), before initiating nutritional support, ensure that the patient's hemodynamic parameters are stable. Subsequently, assess gastrointestinal function using the Acute Gastrointestinal Injury (AGI) grading system. For patients with AGI < Grade III, different initial infusion rates for nutritional support should be used. Patients with AGI Grade IV should discontinue enteral nutrition (EN). For patients at high risk of malnutrition based on their nutritional score, parenteral nutrition (PN) should be initiated; otherwise, PN should be suspended for 7–10 days. Patients receiving EN will be assessed every 4–6 hours using a tolerance score. When the tolerance score exceeds 5 points, EN should be discontinued. Adverse events will be managed and addressed by the treatment team according to standardized protocols. For example, if persistent abdominal pain occurs, enteral nutrition should be discontinued. If deemed necessary by the treatment team, a physical examination and abdominal computed tomography (CT) scan will be arranged. If signs of intestinal obstruction and/or ischemia are present, EN should be immediately discontinued. Diarrhea

may be caused by enteral nutrition, specific diseases, medications, or infections, and the treatment team should consider and diagnose these possibilities.

1. Zhang Z, Li Q, Jiang L, Xie B, Ji X, Lu J, et al. Effectiveness of enteral feeding protocol on clinical outcomes in critically ill patients: a study protocol for before-and-after design. Ann Transl Med. 2016 Aug;4(16):308.
